# Combined transmission, dark field and fluorescence microscopy for intact, 3D tissue analysis of biopsies

**DOI:** 10.1117/1.JBO.25.11.116503

**Published:** 2020-11-19

**Authors:** Marius I. Boamfa, Michel J. A. Asselman, Roland C. M. Vulders, Esther I. Verhoef, Martin E. van Royen, Pieter J. van der Zaag

**Affiliations:** a Philips Research Laboratories, Eindhoven, The Netherlands; b Erasmus MC, Department of Pathology, Rotterdam, The Netherlands; c Erasmus MC, Erasmus Optical Imaging Centre, Rotterdam, The Netherlands

**Keywords:** 3D biopsy imaging;, optical imaging;, transmission microscopy;, bright field imaging;, image processing;, tissue clearing

## Abstract

**Significance:** Currently, tissue biopsies are sectioned into 3- to 5-μm-thick slices that are used for conventional pathology analysis. Previous work by confocal microscopy and light-sheet microscopy has shown that analyzing biopsies intact in three-dimensions (3D) is possible and may lead to a better understanding of cancer growth patterns. Although accurate, these methods require fluorescent staining of the tissue, in addition to tissue clearing. If the 3D biopsy analysis could be done sufficiently swiftly, this approach may be used for on-site assessment of the adequacy of a biopsy taken.

**Aim:** We aim to show that, by transmission microscopy of optically cleared tissue punches, the tissue architecture can be determined without the need for fluorescent staining.

**Approach:** Transmission microscopy is used by combining bright field microscopy with dark field and epifluorescent microscopy to compare samples that have also been analyzed by fluorescent confocal microscopy.

**Results:** With increasing distance to the focal plane, the higher-frequency part of the spatial frequency spectrum of transmitted light is attenuated increasingly. This property is exploited for tissue segmentation, detecting whether tissue is present at a certain position in the focal plane image. Using this approach, we show that a 3D rendering of the internal cavity or tubules structure of punch biopsies, which are up to 1-mm thick, can be acquired in ≈1 min scan time per imaging modality. The images of the overall tissue architecture that are obtained are similar to those from the confocal microscopy benchmark, without requiring fluorescent staining.

**Conclusions:** Images of the overall tissue architecture can be obtained from transmission microcopy; they are similar to those from the confocal microscopy benchmark without requiring fluorescent staining. Tissue clearing is still needed. The total scan time of the present method is significantly shorter at a fraction of the device costs.

## 1 Introduction

In recent years, the interest in the study of cells in their three-dimensional (3D) tissue environment has increased rapidly. This development is fueled by the increase in *in-vivo* imaging methods and the rising demand for more complex 3D model systems for drug testing and development.^1^^–^^3^ To circumvent time-consuming serial sectioning and to avoid misalignment artifacts in 3D reconstruction, several technical advances have been made to obtain 3D information from intact samples using non-linear microscopy approaches such as multiphoton fluorescence microscopy.^4^ In addition, protocols for optical clearing of tissue have been described to improve tissue penetration of light and reduce scattering of the emitted fluorescent signal, increasing the imaging depth by up to several millimeters.^5^^–^^7^ Due to this combination of high-resolution optical techniques and tissue clearing, it has been possible to image deep into biological specimens^8^ and even image whole organs.^9^ These developments have enabled new studies in fundamental research, e.g., embryonic development and neural structures in whole (mouse) brains.^10^^–^^12^ This has led to new insights in developmental biology and neurology.^10^

More recently, non-destructive 3D imaging of clinical samples has been proposed as a potentially valuable diagnostic tool to image intact biopsies^13^ or to assess resection margins in surgical samples (reviewed in Ref. 14). Here, we focus on the imaging of intact prostate tissue punches. Current prostate cancer diagnostics is mainly based on Gleason growth patterns determined from 4- to 5-μm-thin tissue slices.^15^^–^^18^ Despite the clinical importance of these growth patterns, thin sections do not provide the necessary insight in their 3D tissue architecture. Using optical tissue clearing and fluorescent labeling of luminal (CK8/18) and basal (CK5) epithelial cells, distinct 3D architectural structures have been described in different stages of prostate cancer, revealing two major architectural subgroups of prostate cancer growth patterns.^19^^,^^20^ Although this fluorescence confocal microscopy approach provided an important insight in the 3D architecture at cellular resolution, it comes at the cost of speed of acquisition. Using light-sheet microscopy, Glaser et al.^21^ demonstrated a more rapid solution for non-destructive slide-free pathology on optically cleared complete prostate and breast biopsies. In this work, the authors used nuclear (DRAQ5) and cytoplasmic (eosin) fluorescent staining and image conversion to mimic the conventional hematoxylin and eosin (H&E) staining.^21^ Similarly, light-sheet microscopy of cleared solid tumor biopsies identified phenotypic heterogeneity in epithelial-to-mesenchymal transition and angiogenesis, at single cell resolution.^22^ Finally, in immunotherapy, lymphocyte infiltration is a key parameter in assessing therapy effectiveness. Consequently, imaging the tissue and the position of lymphocytes with respect to the cancer cells in a biopsy is important and has been studied in 3D recently.^23^

Here, we report on a system with the unique capability of visualizing the global tissue architecture (e.g., ducts and tubules) in bright field (BF) and dark field (DF), as well as implementing fluorescent detection of cells of interest, which is demonstrated using punches of intact clinical prostate samples.

## 2 Materials and Methods

### 2.1 Clinical Prostate Cancer Samples

Archival formalin-fixed, paraffin-embedded (FFPE) prostate specimens from patients who had undergone radical prostatectomy for prostate cancer at the Erasmus Medical Center between 2012 and 2017 were included. Specimens were fixed in neutral-buffered formalin, transversely cut into 4-mm slices, and embedded in their entirety for histopathologic evaluation. Regions of interest were selected for 3D imaging using H&E-stained tissue sections and included benign hyperplasia, a benign transition zone, and Gleason 4 ill-formed growth pattern with intraductal carcinoma, which is often correlated with aggressive disease. The use of tissue samples for scientific purposes was approved by the institutional Medical Research Ethics Committee (MEC-2011-295, MEC-2011-296) and was in accordance with the “Code for Proper Secondary Use of Human Tissue in The Netherlands” as developed by the Dutch Federation of Medical Scientific Societies (FMWV, version 2002, update 2011).

### 2.2 Tissue Clearing and Immuno-Fluorescent Staining

Punches of 500  μm thickness were collected from regions of the selected FFPE samples with indicated tissue architectures using tissue microarray punching needles (Estigen Tissue Science, Tartu, Estonia). Immuno-fluorescent staining of the intact punches was performed according to an adapted iDISCO protocol.^11^^,^^24^ In short, tissues were dewaxed overnight and subsequently incubated in methanol for 60 min, in 20% dimethylsulphoxide (DMSO) and 20% $H_{2}O_{2}$ in methanol at 4°C, and then overnight in 20% DMSO in methanol. Tissue was rehydrated through a graded methanol, phosphate-buffered saline (PBS) series. This was followed by incubation in a blocking buffer consisting of 0.2% Triton X-100, 10% DMSO, and 0.3 M glycine in PBS. Primary antibodies targeting Keratin 5 and Keratin 8-18 (1:150; EP1601Y; Abcam, Cambridge, UK and 1:75; MS-743; Immunologic, Duiven, The Netherlands) were incubated for 7 days in PBS supplemented with 0.2% Tween20, 10  μg/ml heparin, 5% DMSO, and 1% milk at 37°C. This step was followed by washing in PBS with 0.2% Tween20 and 10  μg/ml heparin. Incubation with secondary fluorescent Alexa 514-labeled and Alexa 647-labeled antibodies (1:200; Life Technologies) was performed in PBS containing 0.2% Tween20, 10  μg/ml heparin, 5% DMSO, and 1% milk for 7 days at 37°C. Prior to clearing, the tissue was washed in 0.2% Tween20 and 10  μg/ml heparin in PBS overnight at 37°C, dehydrated in methanol gradients at room temperature, and subsequently incubated in a mixture of 50% methanol and 50% benzyl alcohol/benzyl benzoate (BABB). Optical transparency was achieved in a 100% BABB solution after 10 min of incubation. After clearing, tissue was stored in BABB at 4°C until imaging. In total, the tissue clearing was done in 3.5 to 4 h, and is thus a 2 orders of magnitude faster process than immuno-staining.

### 2.3 Transmission-Based Microscopy

**Fig. 1:**
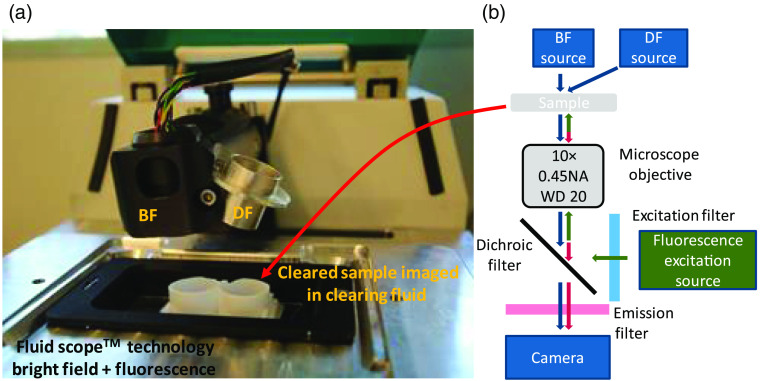
Overview of the setup, showing the BF light source and the DF option. (a) DF imaging is be achieved by simply relocating the light source into the DF option holder. (b) Schematic representation of the setup and light path.

This study uses a modified research version of the oCelloscope device.^25^^,^^26^ This instrument can record images by optical sectioning in epifluorescence mode (FL), BF mode, and dark field (DF) mode at multiple wavelengths [530, 660, and 850 nm for BF and DF and 510 nm (480-nm excitation) and 670 nm (620-nm excitation) for FL]. A multiwavelength source is used for BF illumination, with light impinging almost parallel on the sample (very low NA of the BF condenser). DF uses the same illumination unit, placed at an angle of 30° to the optical axis of the objective to avoid collection of direct light by the objective. The FL mode uses an epifluorescence configuration (Fig. 1).

The instrument is equipped with a microscope objective of 10× magnification (NA 0.45), with a working distance of 20 mm. The field of view (FOV) of the instrument is approximately 1.9×1.1  mm [1920×1080  pixels, CMOS imaging sensor (Fairchild, CIS1910)], with a lateral resolution of 1  μm. The fluorescence filters [Fluorescence filters (Semrock, barrier filter model 733–527/645–25, dichroic mirror model 733–474/23–25, excitation filter model 733–495/605-Di01-25×36] are multiband filters that facilitate multiple wavelength FL modes without mechanical movement of any components. BF and DF illumination light spectrally pass through one of the dichroic and emission filter bands to allow for very fast (<1  ms) switching between imaging modes (DF–FL or BF–FL) using the electronics of the LED current drivers.

Prior to the sample scanning, reference images are acquired for flat field correction for all channels to correct for non-uniformities in illumination for BF and DF and excitation light for FL. In addition, these calibration images eliminate non-uniform camera responses. For fluorescence calibration, we use a fluorescent microscope slide (FSK6, Thorlabs).

Fluorescently labeled and optically cleared samples were placed in a custom-made cartridge^27^ consisting of an open cut syringe barrel mixing cartridge (Nordson EFD, Westlake, Ohio) mounted on a 24×76  mm glass slide (Thermo Scientific, Waltham, Massachusetts) utilizing a multicomponent epoxy adhesive (UHU, Bolton Group, Milan, Italy). The container with the samples submerged in BABB was installed on the microscope slide insert.

**Fig. 2:**
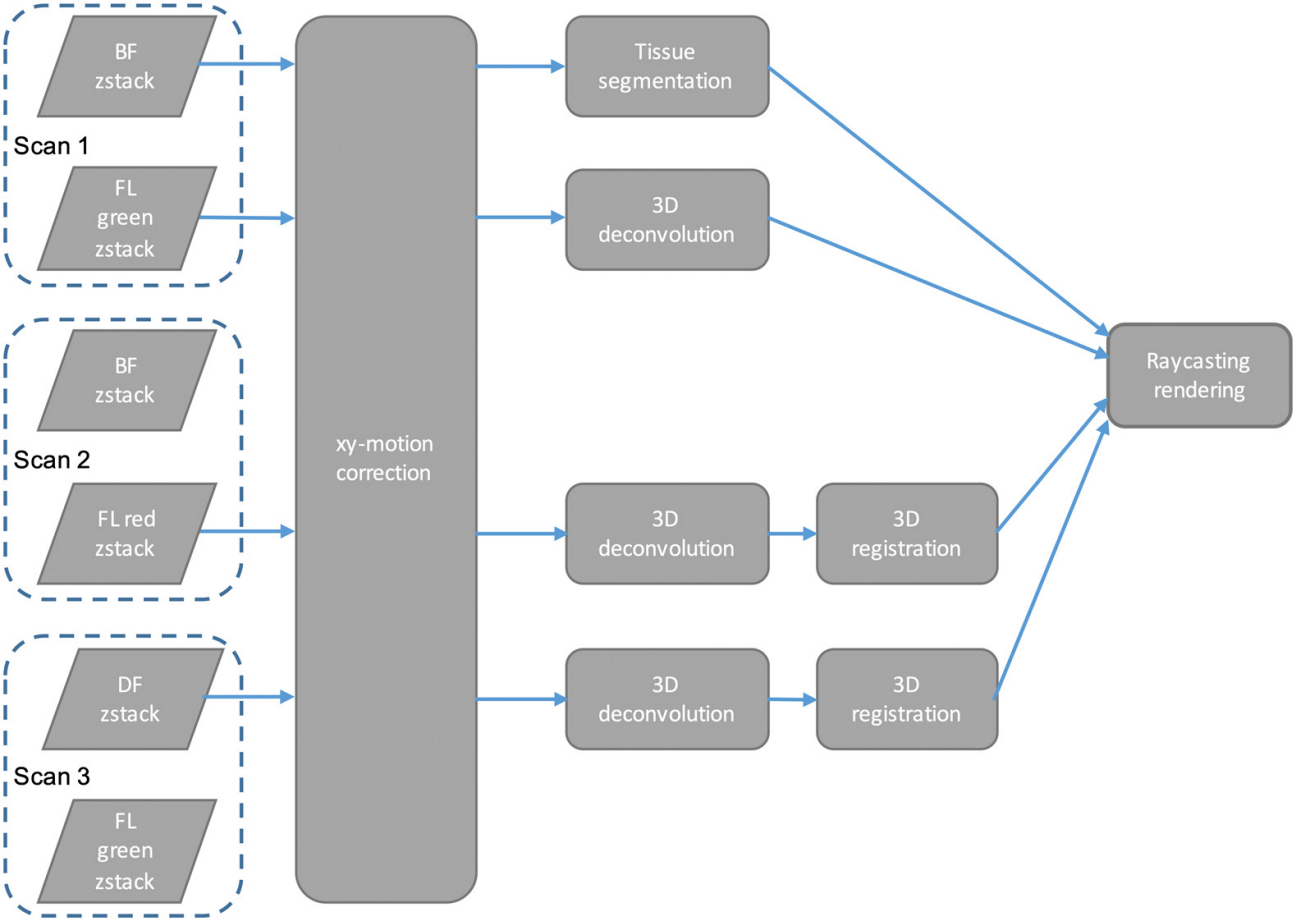
Image processing algorithm.

For each sample, z-stacks comprising 200 focal planes of two sequentially imaged channels (selected from BF, DF, and FL) are obtained to cover the total height of the sample (Fig. 2). During the z-stack acquisition, only the microscope objective (including camera and imaging optics) and the BF/DF condenser are moved, while the sample stage stays fixed to limit movement of the sample within the imaging fluid. The stage is moved only for tiling in the y-direction.

### 2.4 Image Processing

For BF imaging, the scattering of light, due to the mismatch of refraction indices at interfaces between different substances in a tissue, introduces spatial variations in light intensities within the focal plane observed by the scanner. The point-spread function (PSF) describes the response at the image sensor of the imaging system to a point source anywhere in the scanned 3D area. Due to the PSF, points located outside the focal plane will have a contribution to the acquired image. For an increasing distance to the focal plane, the higher-frequency part of the spatial frequency spectrum is attenuated increasingly. This property is exploited for tissue segmentation, detecting if tissue is present at a certain position in the focal plane image. By observing the amount of spatial high-frequent energy present in the spatial local neighborhood of the position in the image, cell tissue is assumed to be present when this energy exceeds a threshold level and tissue is assumed to be absent (e.g., cavities, surrounding medium) when the energy is below the threshold level. By doing this for multiple image positions within multiple focal planes, a complete 3D tissue segmentation of the scanned 3D volume is created. This tissue segmentation has the interesting property that cavities (e.g., tubules) of sufficient size (>50  μm) are detected within the tissue 3D contours in the volume. For FL and DF imaging, the images were deconvolved using the Richardson–Lucy deconvolution algorithm^28^ and an experimental PSF obtained by scanning polystyrene FL beads (3  μm Polybead, Polysciences, Warrington) in glycerol and Triton X100 closely matching the refractive index of the tissue imaging fluid (BABB). This approach was taken because FL beads dissolve in BABB.

To compensate for accidental shift and rotation of the tissue sample in the fluid, the translation between successive focal planes of BF-images is determined using normalized cross correlation and is used to correct the translation in the x- and y-directions of the images by bilinear interpolation. When a biopsy is larger than a single FOV, the complete sample was imaged by tiling multiple overlapping stacks using 3D scale-invariant feature transform^29^ on the BF volume data. Matching landmarks are used to estimate the parameters of the 3D affine transformation. Subsequently, the two volumes are stitched together by applying the affine transform to one of the volumes by doing a tri-linear interpolation and removing the part where both volumes overlap. The four data sets obtained (tissue segmentation, DF, and the two FL channels) are combined by volume rendering.^30^ This has the advantage that all voxels are rendered transparent, and thus occluded structures remain visible.

### 2.5 Confocal Imaging

For imaging, samples were mounted in 100% BABB in glass-bottomed micro-well dishes (MatTek, Ashland, Massachusetts) and covered with a #1 cover glass (Menzel-Glaser, Braunschweig, Germany) to avoid direct contact of the microscope objective and immersion water with the BABB. Imaging was performed with an upright Leica SP5 confocal microscope (Leica Microsystems, Eindhoven, The Netherlands) equipped with a long (1.95 mm) working distance 20× APO water-immersion objective (NA 1.0). Samples were excited by the 514- and 633-nm laser lines from argon and HeNe lasers, respectively. Alexa 514 and Alexa 647 emission light was detected at 525 to 600 nm and 643 to 700-nm, respectively. Z-stacks were recorded with a 3-μm z-step size and a 0.72-μm pixel size. To compensate for loss of signal and optimize the collection of structural information, laser intensity and detector sensitivity were (semi-) automatically adjusted within a preset range. Images were projected in 3D using FIJI (ImageJ 1.49 s^31^).

## 3 Results

### 3.1 Individual Planes form Transmission Microscopy z-Stacks

**Fig. 3:**
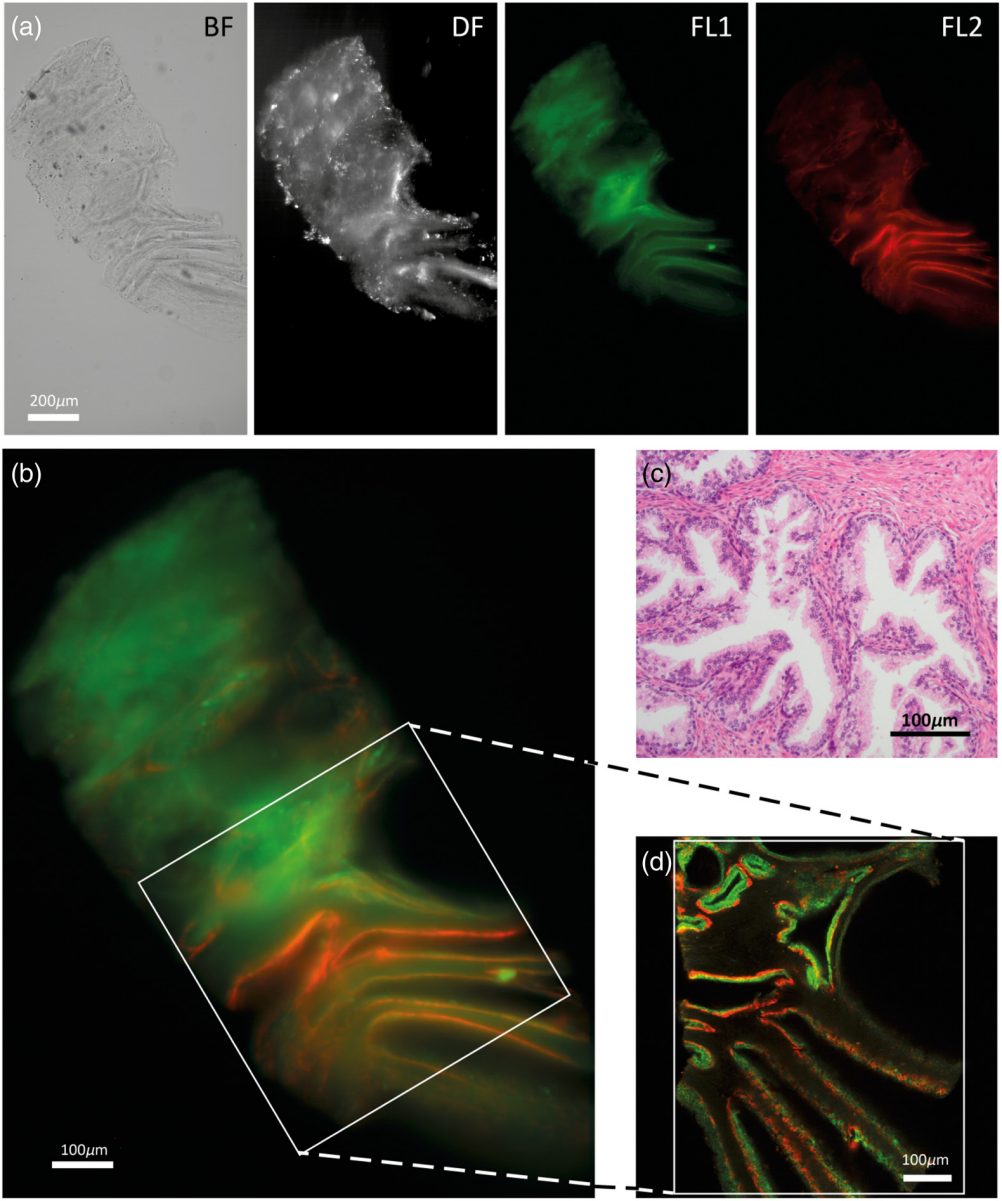
(a) and (b) BF, DF and Alexa488 and Alexa647 Fluorescence (FL1 and FL2, respectively) typical images of the z-stack obtained with the oCelloscope. The scale bar depicted in the right panel represents 200  μm. (c) H&E-stained tissue section, and (d) cross section of the confocal microscope showing the same structures with CK8-18 positive luminal epithelial cells (green) and CK5 positive basal epithelial cells (red) in benign transition zone prostate glands.

Typical tissue biopsies and FFPE punches are 5 to 10 mm in length and have a diameter of 500  μm to 1 mm, which extends the required imaging times due to tiling of adjacent fields in standard confocal microscopy. Due to the tilted imaging, which creates some overlap between sequential images, the transmission microscope enables imaging of intact and optically cleared FFPE prostate tissue punches in 3D without the requirement of serial sectioning, as shown in Fig. 3. As the scale bar shows, a single focal image can cover an area of $2\;{\mathrm{mm}}^{2}$. As the samples examined have an initial thickness of around 500  μm, BABB tissue clearing^5^^–^^7^ was applied to enable penetration of light through the sample both for confocal microscopy^19^ and transmission microscopy. Tissue clearing by BABB leads to shrinkage of (biopsy) samples, as discussed in more detail elsewhere.^27^ A series of about 200 of such images, in tilted z-stack scan, were obtained for each tissue punch studied. The typical dimensions of the samples studied were a circular diameter of 600  μm and a length of about 2 mm. Scanning such a sample could be achieved in about 1 min per imaging channel/modality (BF, DF, and FL). Acquiring all four possible data sets per biopsy requires about 4 min [Fig. 3(a)]. In BF microscopy, contrast is generated by reduction of light in dense tissue regions. Since the contrast is relative to the background of the transmitted light, the contrast in the BF image is low. The DF image, in which the scattering of light by tissue inhomogeneities is the source of the signal, gives much more contrast in comparison. However, the combination of BF and DF enables low-resolution segmentation of the tissue outline and internal structures. The fluorescent signal shows the presence of CK8-18 labeled luminal cells (green) and CK5 epithelial-labeled connective tissue (red) surrounding the prostate glandular structures.

**Fig. 4:**
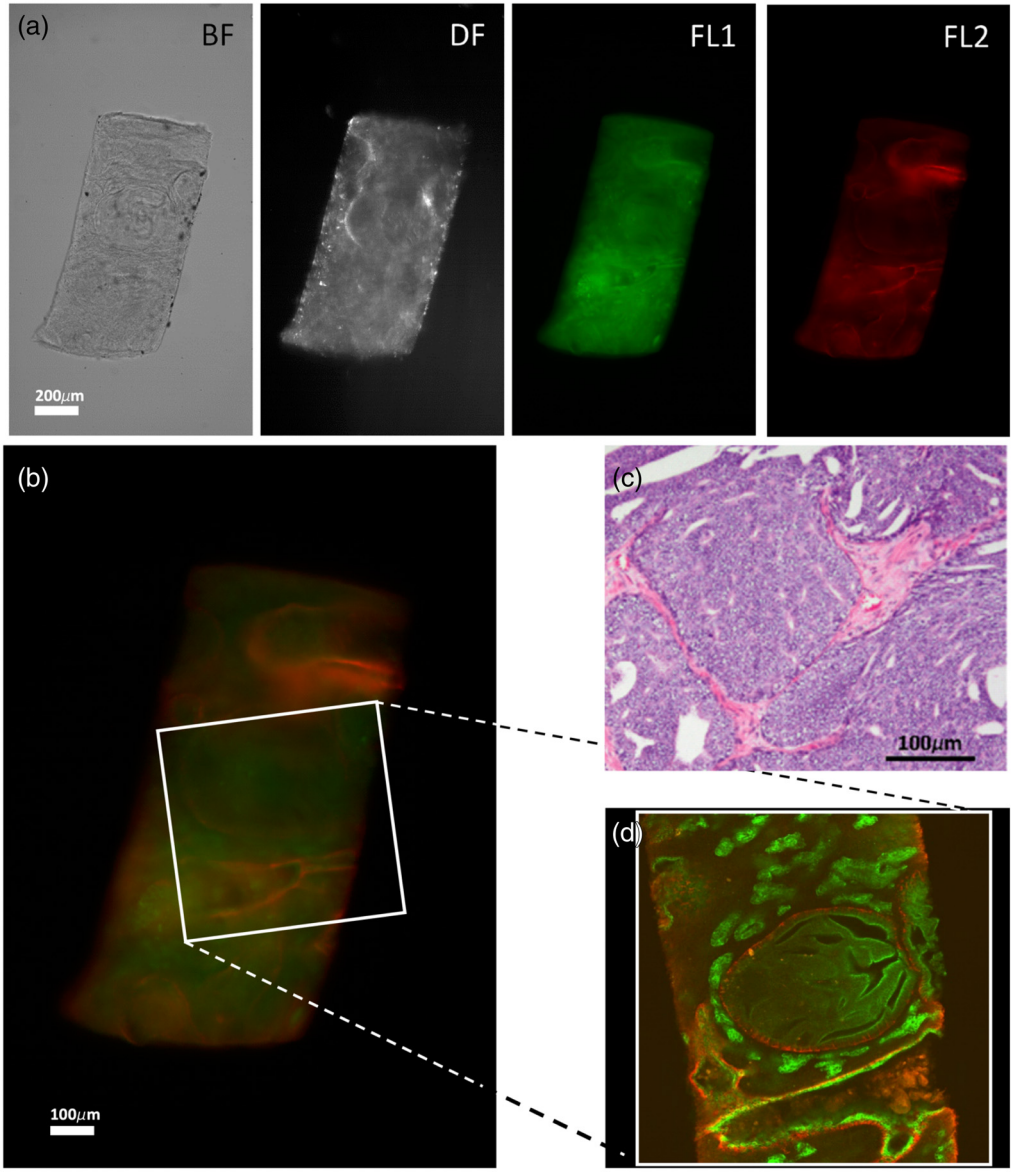
(a) and (b) BF, DF and Alexa488 and Alexa647 Fluorescence (FL1 and FL2, respectively) typical images of the z-stack obtained with the oCelloscope. The scale bar depicted in the right panel represents 200  μm. (c) H&E-stained tissue section, and (d) cross section of the confocal microscope showing the same structures with CK8-18 positive luminal epithelial cells (green) and CK5 positive basal epithelial cells (red) in a Gleason 4 prostate sample with ill-formed growth pattern with intraductal carcinoma.

To assess the validity of the approach, we first compared experimental imaging parameters and resulting 3D images of two clinical prostate tissue punches to confocal microscopy containing either a benign transition zone (Fig. 3) or Gleason 4 prostate cancer tissue with ill formed growth patterns and intraductal carcinoma (Fig. 4). Despite some differences in sample orientation, the same images at similar selected planes show that sample/tissue borders and the glandular structures of prostate tissue could be observed in both samples. These images as well as the fluorescence data are in good agreement, indicating that, with transmission microscopy, the same structures in the tissue images are reproduced as seen in confocal microscopy. An important advantage of the transmission microscopy over laser scanning confocal microscopy is the speed of imaging. A 3-channel stack of 200 frames takes more than 60 min in a laser scanning confocal microscope, whereas a similar stack (with larger FOV) can be obtained within 4 min in the oCelloscope (Table 1). In addition, the transmission microscopy stack includes the DF images, which provide extra information on sample regions with non-uniform optical properties that cause light scattering, such as calcifications or small glandular structures. As a consequence of objectives (20×0.75  NA in the confocal microscope and 10×0.45  NA in the oCelloscope) and detection units (PMT and CMOS camera, respectively) used, the FOV dimensions (x and y) are doubled in the transmission microscope (Figs. 3 and 4). Although this enables covering larger tissue areas per scan, the increase in FOV comes at the cost of lateral resolution: 0.7 μm/pixel in confocal compared with 1  μm/pixel in the oCelloscope.

**Table 1:** System performance compared with other microscopy techniques based on the key optical parameters. This comparison is based on scanning performance only. Note that a typical parameter range is given to reflect that, for each microscopy, a range of systems can be built depending on the optical components chosen.

	Current method	Confocal	Light sheet	TPM
NA	0.4	0.4 to 1.2	0.4 to 1.2	0.4 to 1.2
Lateral resolution (μm)	0.7 to 1.0	0.2 to 0.7	0.2 to 0.7	0.2 to 0.7
Axial resolution (μm)	2.5	0.2 to 2.5	∼1.0	0.2 to 2.5
Depth penetration (mm)	3	∼3	0.3 to 1	∼3
FL and/or BF	BF and FL	FL and possible BF	FL and possible BF	Two-photon FL and SHG
Acquired time 1-mm z stack 400 images	30 s	20 to 40 min	30 s	30 to 60 min
Well plate compatibility	+	+	−	+
Complexity and costs	++	−	−	−−

Quantitative assessment of structural similarity between images is typically done by analyzing metrics such as the structural similarity (SSIM) index^32^ or the mean square error (MSE) (of these, the SSIM better correlates to visual perception of human observers). However, for the confocal microscopy derived images versus the image reconstructions from the transmission microscopy presented here, these analyses are more challenging. Owing to tilting of the 3D sample between the two microscopes, the SSIM values found are 0.4±0.1 between the fluorescent images of both microscopes. This not only expresses the differences introduced by the optical properties of the imaging systems but also mostly the differences in their registration resulting in the inability to select the exact same plane within the sample for the image types.

### 3.2 3D Reconstructed Images

**Fig. 5:**
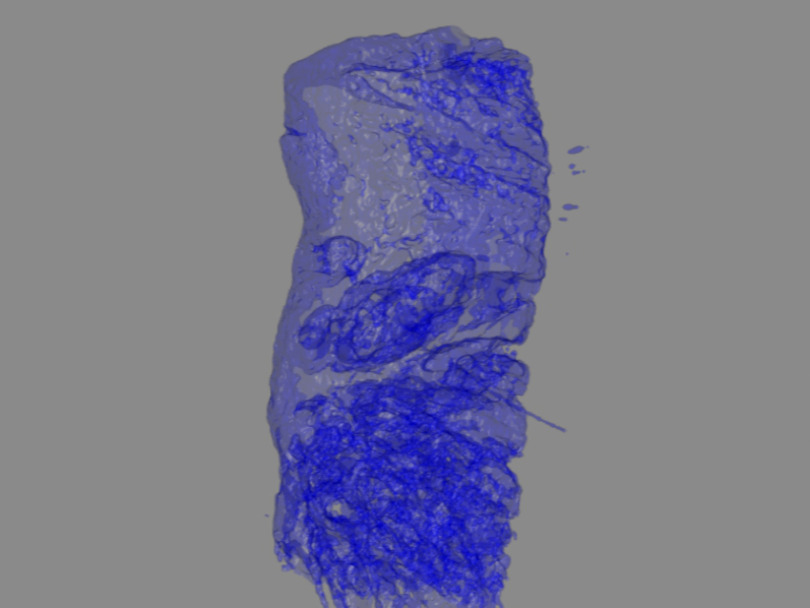
Three-dimensional rendering of the ducts in a prostate sample using BF data and the tissue segmentation algorithm based on the spatial frequency spectrum as discusssed in Sec. 2.4. The ducts are detected in this 900-μm-thick optically cleared prostate biopsy sample. (Video 1, gives the 360° rotation movie of this sample. The clipping of the signal on the sample’s side is due to the biopsy resting on the bottom of the sample container.) (Video 1, mp4, 5324 KB [URL: https://doi.org/10.1117/1.JBO.25.11.116503.1]).

**Fig. 6:**
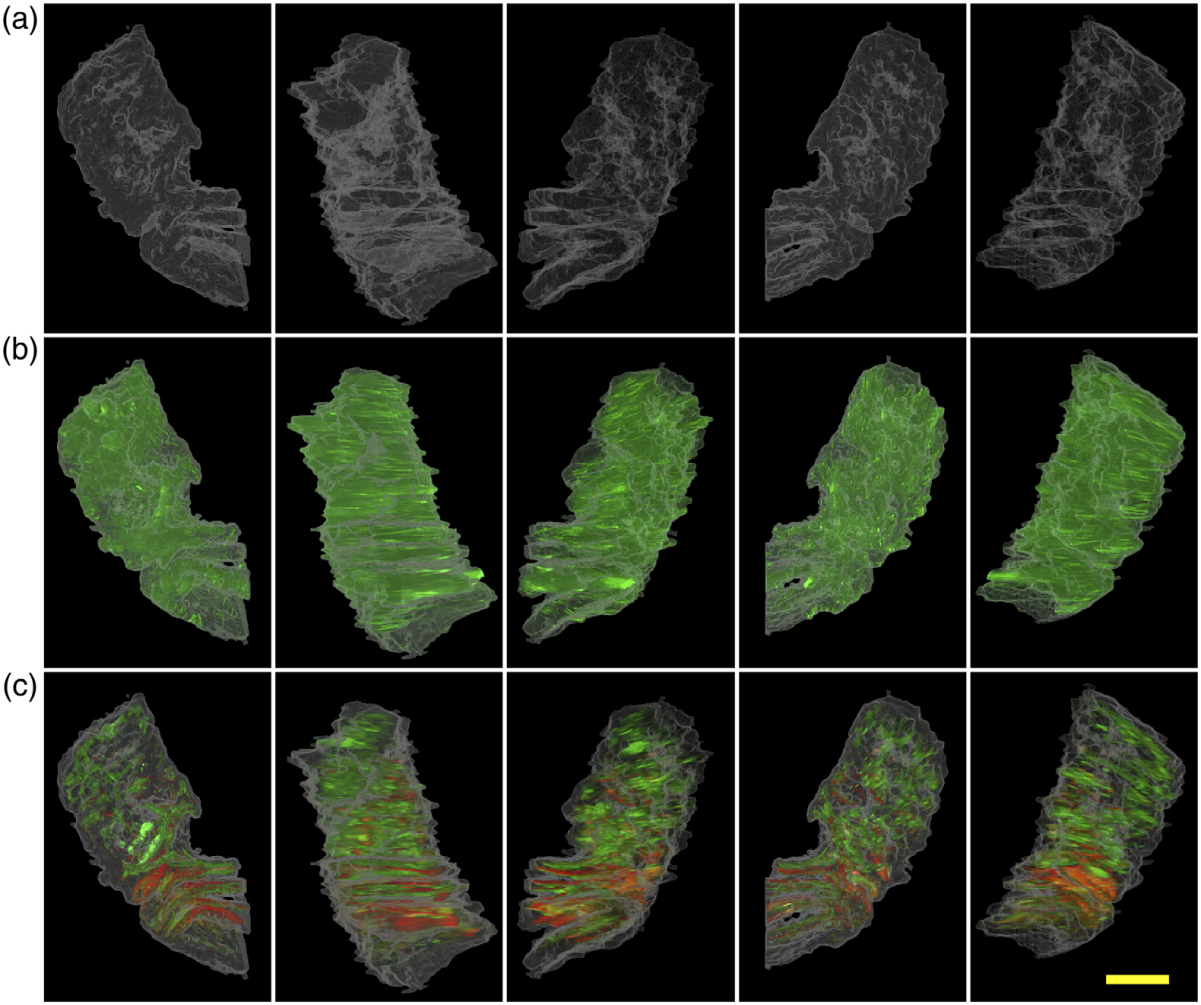
Projection of the ray-casting rendering with the sample in different orientations. (a) Tissue segmentation, (b) tissue segmentation (white) and DF signal (green), and (c) tissue segmentation (white) and two fluorescence channels, CK5 (red), CK 8-18 (green). The scale bar represents 250  μm Movies of the ray-casting renderings are available: Video 2 (top row), Video 3 (middle row) and Video 4 (bottom row). (Video 2, mp4, 1335 KB [URL: https://doi.org/10.1117/1.JBO.25.11.116503.2]; Video 3, mp4, 1339 KB [URL: https://doi.org/10.1117/1.JBO.25.11.116503.3]; Video 4, mp4, 1473 KB [URL: https://doi.org/10.1117/1.JBO.25.11.116503.4]).

As the previous section has shown, a relevant advantage of transmission microscopy is that the information about tubules can be obtained without staining the samples, whereas in confocal microscopy this depends on fluorescent staining. The BF information shows variations in tissue architecture based on scattering properties and is used to create a 3D map of the biopsy morphology, including internal cavities and tubules (Fig. 5). In the next step, this 3D map is used to position the information contained by the DF channel and the two fluorescence channels (Fig. 6). The result is a 3D rendering, wherein the sample borders and the internal sample structure are clearly defined, and herein the specific information obtained from the fluorescence channels and DF channel are projected. Without the use of ray-casting, the internal structures would be obscured by the external sample border. Figure 6 illustrates the results of the image processing algorithms in different angle projections of the 3D volume. The complete films of the sample rotations are available (see Video 2, Video 3, and Video 4) for tissue segmentation, tissue segmentation and DF information, and tissue segmentation plus the two fluorescence channels.

The data show that it is possible to reconstruct a 3D image of the biopsy, having morphological information based on BF [Figs. 5 and 6 (top row)]. The internal structure of the sample can be visualized. Detecting the ducts may be a useful first step in screening for malignant glands in unsectioned prostate biopsies.

## 4 Discussion

In the literature, several methods have been described for the analysis of intact biopsies in 3D, including the use of confocal microscopy,^19^^–^^21^ two-photon microscopy,^4^ and light-sheet microscopy.^21^^,^^22^ The transmission microscopy approach presented here differentiates itself from these methods in various aspects, each with their benefits and limitations. See Table 1 for an overview of typical parameter ranges at which these microscopies operate for 3D biopsy imaging.^4^^,^^19^^–^^22^^,^^24^ In this comparison, we compared the imaging aspects since the preparation of the biopsy sample through tissue clearing is needed for all of these optical methods. Of these imaging modalities, confocal and light-sheet microscopy typically rely on operating in fluorescence mode, while the current technique images the sample in BF and DF modes in addition to the FL mode. The BF and DF images show inhomogeneities in tissue architecture independent of fluorescent labeling, in which the added value of DF is the visualization of the overall sample morphology. The comparatively fast imaging of transmission microscopy in providing an overview of the sample as a whole comes at the expense of resolution. Both lateral and axial resolutions depend on the optics (objective and detector) used. The lateral resolution is comparable for all three methods (dependent of the NA of the imaging lens and illumination optics). Yet, the axial resolution is better for the confocal and light-sheet microscopy techniques. In the next steps, the axial resolution could be improved by adding an additional scan at an angle of 90°, which is subsequently processed together with the others. Finally, transmission-based microscopy is a cost-effective alternative as its costs about an order of magnitude less than either confocal or light-sheet microscopy.

Overall, the analysis of intact biopsies by optical means in 3D may be used to gain further insights in tumor growth patterns over medical imaging as optical imaging offers a 3 orders of magnitude higher resolution (μm versus mm). In future coupling, the current 3D pathological analysis to medical imaging information will enable obtaining more detailed information on local tumor growth. In addition, rapid assessment of whether an adequate biopsy has been obtained could reduce potentially the number of invasive biopsies taken. For the latter clinical application, short processing times are essential. This most likely excludes conventional confocal microscopy, in which scanning time is limited by the pixel dwell time and resulting scan speed. The scanning speed of confocal microscopy can be improved using spinning disk technology.^33^^,^^34^ However, this comes at the expense of optical signal loss and hence penetration depth. By contrast, imaging times of transmission microscopy are only limited by the z-axis actuator/motor. In the current system, complete tissue punches and most likely clinical biopsies can be imaged within minutes, which is on par with light-sheet microscopy. However, this will require a sufficiently fast tissue clearing process as all optical methods require tissue clearing for sufficient light penetration deep within the tissue. Since BF and DF do not require additional (immuno)-staining, these preprocessing times can be limited to hours rather than days. Faster tissue clearing protocols would obviously be of benefit.

## 5 Conclusion

We have shown that combining transmission microscopy (BF and DF) with an epifluorescence system enables fast (5 min) 3D biopsy visualization. Through the detection of the spatial high-frequency energy from BF images, the internal cavity or tubules structure can be detected without requiring fluorescent staining. We have shown that images of the overall tissue architecture that are similar to those from the confocal microscopy benchmark can be obtained. Tissue clearing is still needed. Furthermore, the total scan time of the present method is significantly shorter at a fraction of the costs. If a faster tissue clearing method would become available, this may open the way to fast 3D assessment of intact prostate biopsies and tissue punches. Finally, we have shown that DF imaging offers additional contrast to BF imaging.
