# Supplementary material for: Combined transmission, dark field and fluorescence microscopy for intact, 3D tissue analysis of biopsies
Source: J Biomed Opt. 2020 Nov 19;25(11):116503. doi: 10.1117/1.JBO.25.11.116503 (PMC7676494; doi:10.1117/1.JBO.25.11.116503)
Supplement: Supplementary file 1 [file JBO_025_116503_SD001.docx]

# (Supplementary information)

# Title

Combined Transmission, Dark field and Fluorescence Microscopy for Intact, 3D Tissue Analysis of Biopsies.

# Authors

Marius I. Boamfa ^1^, Michel J.A. Asselman ^1^, Roland C.M. Vulders^1^, Esther I. Verhoef ^2^, Martin E. van Royen^2,3^  and Pieter J. van der Zaag ^1^

# Author affiliations

^1^ Philips Research laboratories, High Tech Campus 11, 5656 AE, Eindhoven, The Netherlands.

^2^ Erasmus MC, Department of Pathology, Wytemaweg 80, 3015 CN Rotterdam, The Netherlands.

^3^ Erasmus MC, Erasmus Optical Imaging Centre, Wytemaweg 80, 3015 CN Rotterdam, The Netherlands.

# Keywords

3D biopsy imaging, optical imaging, transmission microscopy, bright field imaging, image processing, tissue clearing

With this manuscript the following supplementary information is provided of movies of 3D renderings shown in the manuscript.

Video S1

Movie (360^0^ rotation) of the three dimensional rendering of the ducts in a benig hyperplasia prostate sample using bright field data and the tissue segmentation algorithm based on the spatial frequency spectrum as discusssed in section 2.4. The ducts are detected in this 900 µm thick optically cleared prostate biopsy sample, see Fig. 5. The clipping of the signal of the sample side is due to the sample resting on the bottom of the sample container.

Video S2

Three movies of 3D ray-casting rendering of the sample. A: tissue segmentation; B: tissue segmentation (white) and dark field signal (green); C: tissue segmentation (white) and two fluorescence channels, CK5 (red), CK 8-18 (green), see Fig. 6.
